# Matrix metalloproteinase-2 and pH-responsive drug eluting multilayer as intraocular lens coating to improve the posterior capsule opacification inhibition

**DOI:** 10.1093/rb/rbaf077

**Published:** 2025-07-28

**Authors:** Yuemei Han, Jiahao Wang, Hao Chen, Quankui Lin

**Affiliations:** National Engineering Research Center of Ophthalmology and Optometry, School of Biomedical Engineering, School of Ophthalmology and Optometry, Eye Hospital, Wenzhou Medical University, Wenzhou 325027, China; National Engineering Research Center of Ophthalmology and Optometry, School of Biomedical Engineering, School of Ophthalmology and Optometry, Eye Hospital, Wenzhou Medical University, Wenzhou 325027, China; National Engineering Research Center of Ophthalmology and Optometry, School of Biomedical Engineering, School of Ophthalmology and Optometry, Eye Hospital, Wenzhou Medical University, Wenzhou 325027, China; National Engineering Research Center of Ophthalmology and Optometry, School of Biomedical Engineering, School of Ophthalmology and Optometry, Eye Hospital, Wenzhou Medical University, Wenzhou 325027, China

**Keywords:** posterior capsule opacification, intraocular lens, stimuli-responsive, drug-eluting coating, polysaccharide multilayer

## Abstract

Intraocular lens (IOL) is a crucial implant for cataract therapy. Posterior capsule opacification (PCO) is the most common postoperative complication after IOL implantation, which is the abnormal hyperplasia of the residual lens epithelial cells (LECs) after IOL implantation in cataract surgery. It is reported that the cellular microenvironment in the lens capsule changes after surgery, such as the elevated secretion of matrix metalloproteinases (MMPs) and a decrease in pH due to undesired cell proliferation. In this study, MMP-2 and pH-triggered drug delivery polysaccharide multilayer coating was designed and introduced onto the IOL surface for obtaining the cellular microenvironment-sensitive drug-eluting intraocular implant. The methacrylated heparin (HEP-MA) was synthesized and used to layer-by-layer self-assemble with the doxorubicin-loaded chitosan nanoparticles on the IOL surface. The matrix metalloproteinase-2 (MMP-2) sensitive peptide with cysteine contained in both ends (GCRD-GPQGIWGQ-DRCG) was then used to crosslink the polysaccharide multilayer via the Michael addition reaction between sulfhydryl group in cysteines and double bonds in methacrylate groups. The multilayer construction and subsequent cross-linking were validated through ultraviolet–visible spectrophotometer (UV–Vis) and Fourier transform infrared spectroscopy (FTIR). After modification, the IOL material surface becomes more hydrophilic while the optical properties were well maintained. The MMP-2 and pH-sensitive drug sustained-release coating were successfully obtained on the IOL surface via such design. The enzyme-triggered cell proliferation inhibition was realized in the *in vitro* experiments. In an animal model, significant up-regulation of MMP-2 was observed in the aqueous humor after cataract surgery. The multi-functionalized polysaccharide-coated IOL implanted in the animal eye via cataract surgery effectively inhibits PCO formation while it keeps good *in vivo* biosafety.

## Graphical abstract

**Figure:**
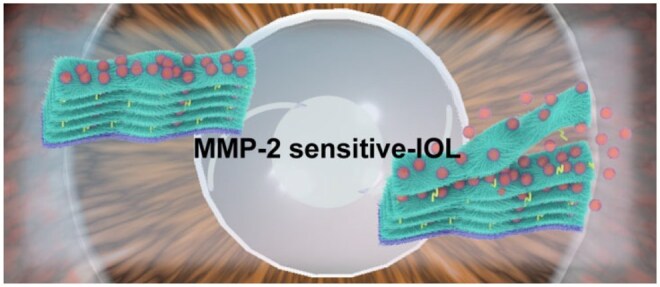

## Introduction

With the aging of the population, age-related cataract has become the most important reversible blindness disease worldwide. It is reported that about 95 million people worldwide are affected by the cataract [1, 2]. Although some eye drops have been investigated to reduce or prevent the cataract in basic research, surgical intervention is still the only effective cataract therapy clinically [3]. The opaque lens was removed and an intraocular lens (IOL) was implanted into the capsular bag in the surgery. However, posterior capsule opacification (PCO), a common postoperative complication, happens in high incidence after the IOL implantation. It causes the posterior capsule turbidity and leads to light scatter which reduce the visual quality seriously [4–6]. PCO is related to the proliferation of postoperatively residual lens epithelial cells (LECs) in the capsular bag [7, 8]. Various strategies have been employed to decrease the incidence of PCO, including surgical technique improvement, IOL design optimization, novel IOL material investigation, as well as the pharmacological administration to suppress LECs proliferation during the surgery [9–11]. Nevertheless, PCO has not been eradicated. The PCO incidence is still around 8–34.3% in adults [12], and almost 100% in children [13]. Mild PCO requires neodymium-doped yttrium aluminum garnet (Nd:YAG) laser treatment, whereas severe PCO may need surgery again, which requires additional costs and may also lead to some additional complications such as IOL injury and macular edema [14]. Exploring a safe and effective method to eliminate residual LECs remains a critical challenge.

**Figure 1.**
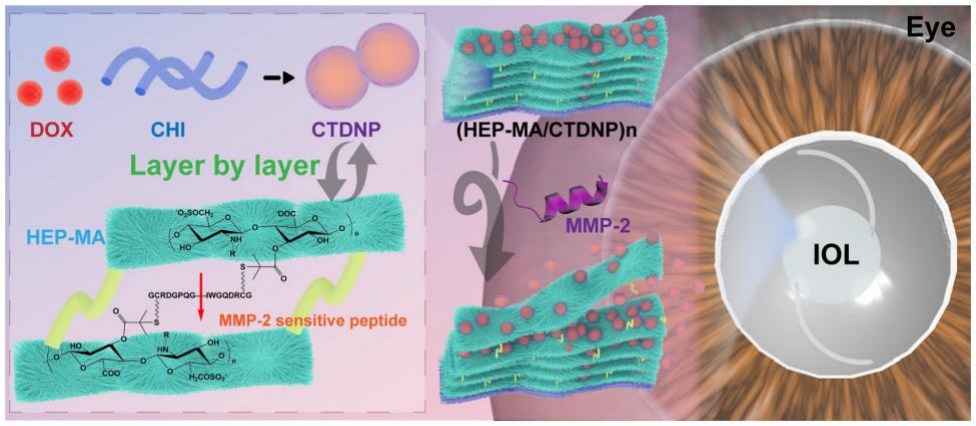
Schematic diagram of HEP-MA/CTDNP surface modification on IOL and MMP-2/pH-triggered drug release for posterior capsule opacification inhibition.

The smart drug delivery system provides a possible way to achieve it. It is reported that the cellular microenvironment in the lens capsule is changed after the surgery, such as the elevated matrix metalloproteinases (MMPs) secretion and the decreasing pH due to undesired cell proliferation [15, 16]. Studies have shown that the matrix metalloproteinase-2 (MMP-2) expression is significantly up-regulated in the eye after cataract surgery [17]. In our previous studies, anti-proliferative drug-loaded polyelectrolyte multilayers can be fabricated for IOL surface modification to inhibit PCO development [18–20]. On the other hand, the enzyme-responsive drug release system has the characteristics of mild reaction conditions, low toxicity, high specificity and low-level expression in normal tissues but high-level expression in specific tissues [21, 22]. Herein, the enzyme and pH stimulus-responsively drug release coating was designed and fabricated on the basis of previous drug-loaded polysaccharide multilayers [23], for the purpose of obtaining a cellular microenvironment-sensitive drug eluting multilayer as IOLs surface coating for smart PCO prevention. Similar to the previous studies, the anti-proliferative drug-loaded cationic chitosan nanoparticle was fabricated and deposited onto the IOL surface by layer-by-layer electrostatic self-assemble with anionic polysaccharide heparin (HEP), endowing the IOLs with excellent anti-adhesion and proliferation properties [24–26]. In particular, the HEP was functionalized by the methacrylate (MA) groups by methacryloylation in advance. The MMP-2 sensitive peptide with cysteine contained in both ends (GCRD-GPQGIWGQ-DRCG) was then used to crosslink the polysaccharide multilayer via the Michael addition reaction between sulfhydryl group in cysteines and double bonds in methacrylate groups. It is hypothesized that the incorporated anti-proliferative drug in the multi-functionalized multilayer coating will be, responsively, released when triggered by the cellular microenvironment alteration when the residual LECs proliferation, such as up-regulation of the MMP-2 or the decrease of pH. Compared to previous studies, this stimuli-responsive drug-eluting coating may enhance *in vivo* biosafety while maintaining excellent PCO inhibition effects (see Figure 1).

## Materials and methods

### Materials

Polyethylenimine (PEI, average Mn = 25 000), methacrylic anhydride (AMA), dimethylformamide (DMF), chitosan (CHI, molecular weight around 179.17 kDa, high viscosity (>400 mPa s)), photo-initiator 2959 (I2959), Fluorescein Diacetate (FDA), N-hydroxysulfosuccinimide sodium (NHS) and N-(3-(dimethylamino)propyl)-N′-ethyl carbodiimide hydrochloride (EDC) were purchased from Sigma-Aldrich LLC. 4-(2-Hydroxyerhyl) piperazine-1-erhaesulfonic acid (HEPES), sodium tripolyphosphate (TPP) and HEP were purchased from Aladdin Biochemical Technology Co., Ltd. MMP-2 sensitive (GCRD-GPQGIWGQ-DRCG) and insensitive (GCRD-GDQGIAGF-DRCG) peptide sequences were provided by Sangon Biotechnology Co., Ltd. Doxorubicin hydrochloride (DOX) was purchased from Dalian Meilun Biotechnology Co., Ltd. Fetal bovine serum (FBS), DMEM/F12 (1:1) cell culture media and 0.05% trypsin-EDTA were brought from Invitrogen. The human lens epithelial cell line (HLEB3) was originated from the American Type Culture Collection (ATCC). Commercialized foldable hydrophobic acrylic IOLs (the optical zone diameter is 6 mm) were supplied by 66 Vision Technology Co., Ltd. Rabbit matrix metalloproteinase-2 (MMP-2) ELISA Kit was purchased from Shanghai Keshun Biological Technology Co., Ltd. The commercialized clinical postoperative administration drugs were obtained from the Eye Hospital of Wenzhou Medical University. All other chemicals were of analytical grade and used without further purification.

### Chitosan nanoparticle preparation and HEP-MA synthesis

The DOX-loaded chitosan nanoparticle (CTDNP) was prepared by the same method as our previous investigations [23]. The detailed characterization of CTDNP is shown in the Supplementary Information. For HEP-MA synthesis, 0.20 g heparin was dissolved in 10 mL ultrapure water. After 6.7 mL dimethylformamide (DMF) was dropwisely added into the HEP solution (v/v = 3/2, water/DMF), 0.154 g (1.5 mmol) methacrylic anhydride (2 molar equivalents relative to the moles in HEP repeating units) was added under stirring. The reaction was maintained overnight at 4°C and the pH of the reaction mixture was maintained at 8–9 with 0.5 M NaOH. The reaction was stopped by adding 0.5 M NaCl solution. The products were precipitated in the water/alcohol mixture solvent (v/v = 2/3). The precipitate was collected and rinsed by the gradient water/alcohol mixture solvent (3/7, 1/4, 1/9, v/v) sequentially. The product was then dissolved in ultrapure water and purified by dialysis (MWCO = 1000 Da). After lyophilization, the final product (HEP-MA) was obtained and stored at 4°C. For proton-nuclear magnetic resonance (^1^H NMR) analysis, the HEP-MA was dissolved in deuterated water (10 mg/mL).

### Enzyme-sensitive drug eluting multilayer coating fabrication

The preparation process of the multilayer film is similar to the previous work [27, 28]. The IOL material was ultrasonically cleaned in pure water for 5 min and dried with a stream of nitrogen. Then IOL material was immersed in 3 mg/mL PEI solution for 2 h to bring the amino groups onto the surface, followed by the same wash and dry processes. Then, the positively charged IOL material was immersed in 1 mg/mL HEP-MA (dissolved in NaAc buffer solution) for 15 min, and the sample was rinsed twice with NaAc buffer and nitrogen dried, obtaining negatively charged HEP-MA adsorbed surface. The negatively charged IOL material was then immersed in the CTDNP solution for another 15 min and washed twice with 1% acetic acid solution (v/v), obtaining a surface with CDNPD coating. Thus a (HEP-MA/CTDNP) bilayer coating was obtained. The deposition cycle of HEP-MA and CTDNP was repeated until the desired number of bilayers was obtained [29, 30]. Furthermore, cysteine contained MMP-2 sensitive peptide sequence (GCRD-GPQGIWGQ-DRCG) was used to crosslink the (HEP-MA/CTDNP) multilayer coating via the Michael addition reaction between methacrylate groups in HEP-MA and sulfhydryl groups in cysteine. Briefly, (HEP-MA/CTDNP) multilayer coating-modified materials were immersed in the peptide solution (2 mg/mL) containing photo-initiator I2959 (0.5 mg/mL) overnight to facilitate the peptide diffusion into the multilayer and Michael addition reaction cross-linking. Finally, the multilayer coating-modified materials were irradiated with 365 nm ultraviolet light (30 W) for 30 min to enhance the cross-linking reaction. The layer-by-layer deposition process was followed by ultraviolet–visible (UV–Vis) spectra and the morphologies of multilayered surface with different bilayer numbers were observed by the scanning electron microscope (SEM). The multilayer cross-linking was confirmed by Fourier transform infrared spectrum (ATR-FTIR).

### Coating properties investigation

The surface wettability of modified materials was measured by water contact angle (WCA) analysis [31, 32]. Water drops with a volume of 2 μL were dropped onto the surface and the static WCA was calculated with the software.

### (HEP-MA/CTDNP)_10_-IOL optical performance detection

Modulation transfer function (MTF) reflects the contrast change of the image, which is more accurate for judging the imaging properties and contains more comprehensive information [33]. Therefore, it is more convincing to describe the image quality of the human eye according to the change of MTF with spatial frequency. Fourier’s theory believes that all images can be regarded as composed of spatial gratings with different frequencies of light intensity distributed according to sine waves. The brightness distribution of the object is the input, and the brightness distribution of the image is the output. It can be calculated with MTF = [M image (*N*)]/[M object (*N*)]. M image represents the contrast of the image, and M object is the contrast of the object. *N* represents the spatial frequency. MTF ≥ 0.43 is qualified. The optical properties of IOL are very important, so the modified IOL needs to satisfy that its optical properties are not affected. We tested the change of MTF of IOL before and after surface modification.

### Enzyme-triggered drug release investigation

In the drug release experiment, the collagenase IV was dissolved in Hanks solution (0.5 mg/mL) served as enzyme contained releasing solution. The pH of the releasing solution was adjusted to 5.5 for simulating the pathological conditions or 7.4 for simulating the physiological environment. Briefly, the samples were put into glass bottles. Then 3 mL release solutions including non-responsive environment (Hanks solution, pH = 7.4), pathological conditions (Hanks solution, pH = 5.5), pure enzyme-responsive environment (collagenase solution, pH = 7.4), pH and enzyme-responsive environment (collagenase solution, pH = 5.5) were added to each bottle, respectively. The drug release experiment was performed on a constant temperature shaker at 37°C. About 0.5 mL release solution was taken out for test at determined time points, and 0.5 mL fresh release solution was added into the bottles. The released solution was measured with a fluorescence spectrometer, each sample was provided in triplicate. The excitation wavelength (*λ*_ex_) was 490 nm and the emission wavelength (*λ*_em_) was 590 nm. Cumulative drug release was calculated according to the standard curve.

### *In vitro* cell experiment

(HEP-MA/CTDNP)_10_-IOL material and unmodified materials were sterilized and placed in 96-well plates, the HLEB3 were harvested by trypsinization, then seeded at 1 × 10^5^ cells/well in DMEM medium supplemented with 50 μg/mL collagenase or Hanks solution, respectively. After 24 h of incubation, the cells were stained with FDA and photographed with a fluorescence microscope [34]. Statistical analysis with single-factor analysis was adopted after cell count (Image J, National Institutes of Health, USA) to analyse the effect of anti-adhesion and anti-proliferation of LECs on modified and unmodified IOL material.

### *In vivo* investigation

#### MMP-2 expression

MMP-2 participates in extracellular matrix (ECM) remodeling after wounding of the corneal surface. In order to detect the changes of MMP-2 expression in the eyes after cataract surgery, we measured the level of MMP-2 in the aqueous humor of animals with and without surgery. Briefly, rabbits (Japanese white rabbits, male) of 2 months old were included in this experiment. There are three rabbits in each group. The right eyes were set as the eyes for cataract surgery. Phacoemulsification cataract surgery was performed by an experienced ophthalmologist. The surgical procedure was the same as our previous investigations [23], performed in accordance with standard cataract surgical procedures proposed by the Association for Research in Vision and Ophthalmology (ARVO). The animal experiments were approved by the Committee and Laboratory Animal Center of Eye Hospital, Wenzhou Medical University. The ethical approval number was YSG25020802. Briefly, after a 2.2 mm temporal corneal incision was made, ophthalmic viscoelastic agent was injected and a continuous curvilinear capsulorhexis (CCC) was subsequently created to allow hydro-dissection and phacoemulsification. Then, the folded IOLs were implanted into the capsular bag with a dedicated IOL injector. After surface anesthesia, aqueous humors in the right eyes were collected as the experimental group, and aqueous humors collected from the left eyes were served as the control group simultaneously at the third week after the operation. The aqueous humors were stored in the refrigerator at −80°C and processed for Enzyme-Linked Immunosorbent Assay (ELISA) as needed. The standardized products were dissolved 15 min before use and gradually diluted to 20, 40, 80, 160 and 320 ng/mL, respectively, the standard dilution was used as the blank control group. ELISA standard curves with different concentrations were obtained. Data are reported as mean ± standard deviation (*n* = 3). The samples were diluted five times before use and set three replicate wells. After optical density (OD) at 450 nm was read with a fluorescence spectrometer, the sample concentration was calculated according to standard curves.

### *In vivo* experiment

The (HEP-MA/CTDNP)_10_-IOL and the unmodified IOL (control group) were implanted into the animal eyes through phacoemulsification combined with IOL implantation. Briefly, 10 Japanese white rabbits (male) were randomly divided into two groups (five rabbits in each group). The right eyes in each group were used to implant the (HEP-MA/CTDNP)_10_-IOL and the control-IOL, respectively. Rabbits were observed, and assessed in accordance with the guidelines of the Society for Research in Vision and Ophthalmology. Congenital eye diseases in the rabbits were excluded through slit-lamp examinations. The IOLs (both unmodified and modified IOLs) were sterilized with ethylene oxide. The surgical procedure was the same as above. The eyes were dilated and evaluated by slit-lamp microscopy observation postoperatively. Anterior section photos were taken for evaluation of PCO development, anterior chamber exudation, and inflammation. The intraocular pressure (IOP) was measured on the third day and first, second, third, fourth and fifth weeks after implantation. Ocular corneal endothelium was observed by specular microscopy at the fifth week.

### Histological experiment

Animals were euthanized in the fifth week after over anesthesia. The eyeballs were taken out and fixed in 4% paraformaldehyde for histological analysis. The cornea, iris, retina and lens capsular bag of the eyeball were separated, and the lens capsular bag was observed and photographed with a stereomicroscope to evaluate the PCO. A gross examination (Miyake-Apple View) of the capsular bag was performed to assess the severity of PCO. PCO is divided into three areas, central PCO (CPCO, the capsule areas behind the IOL optical center with a diameter of 3.0 mm), peripheral PCO (PPCO, the capsule areas behind the periphery of the IOL optic, except CPCP area) and Soemmering’s ring (SR) formation (equatorial region of the capsular bag outside the IOL optic area) [35, 36]. The severities of PCO were scored. The PCO classification ranges from mild, moderate to severe are scored to be 1–3, respectively. The independent sample *t*-test was performed on CPCO, PPCO and SRPCO scores to compare the severity of PCO between the two groups.

Other intraocular tissues were fixed for histopathology staining to evaluate the PCO severity and biocompatibility via the ocular tissue morphology observation and posterior capsule hyperplasia thickness calculation. Histopathology methods include hematoxylin-eosin (HE) and immunofluorescence. The fixed tissues were cut into slices with a thickness of 5 μm for HE staining, the nucleus of LECs can be stained blue, the cytoplasm can be stained red and the ECM secreted by LECs in the capsular bag can be stained red. Immunofluorescence was used to evaluate PCO by staining the capsule membranes to observe the number and morphology of cells on the capsule. The rhodamine isothiocyanate (RITC)-labeled phalloidin was chosen to stain the cytoskeleton, and 4′,6-diamidino-2-Phenylindole (DAPI) was used to stain the nucleus. The confocal microscope was used for observation. The process is shown as follows. Firstly, capsule was carefully separated into the anterior capsule and posterior capsule, and placed on a glass slide. After being washed three times with PBS for 10 min, the cell membrane was destroyed with 0.1% Triton X-100 for 3 min at room temperature, and the capsule was washed with PBS for three times. About 5 μL of RITC-Phalloidin stock solution was added to 1 mL of PBS to make a working solution for staining the cytoskeleton and staining for 40 min at room temperature. To prevent evaporation, the staining process was performed in a closed wet box. RITC-Phalloidin is used at a concentration of 5 μg/mL. To reduce nonspecific staining, 1% bovine serum albumin was added to PBS when preparing the RITC-Phalloidin working solution. The cells are washed with PBS three times. Finally, the DAPI-containing anti-fluorescence quenching mounting solution is added, and the cover glass is sealed with nail polish.

### Statistical analysis

The results were expressed as mean ± SD. Each experiment was repeated independently at least three times. The statistical comparisons of MMP-2 concentration in aqueous humor, IOP and severity of PCO scores in the (HEP-MA/CTDNP)_10_-IOL and control-IOL groups were carried out using independent sample *t*-tests. Statistical comparison of *in vitro* cell experiments was carried out using ANOVA (analysis of variance) with an LSD test. The SPSS 21 (IBM, Armonk, NY, USA) statistics software was employed to perform all of the statistical analyses. The value of *P *< 0.05 was considered significant.

## Results

### (HEP-MA/CTDNP)_10_-IOL characterization

**Figure 2.**
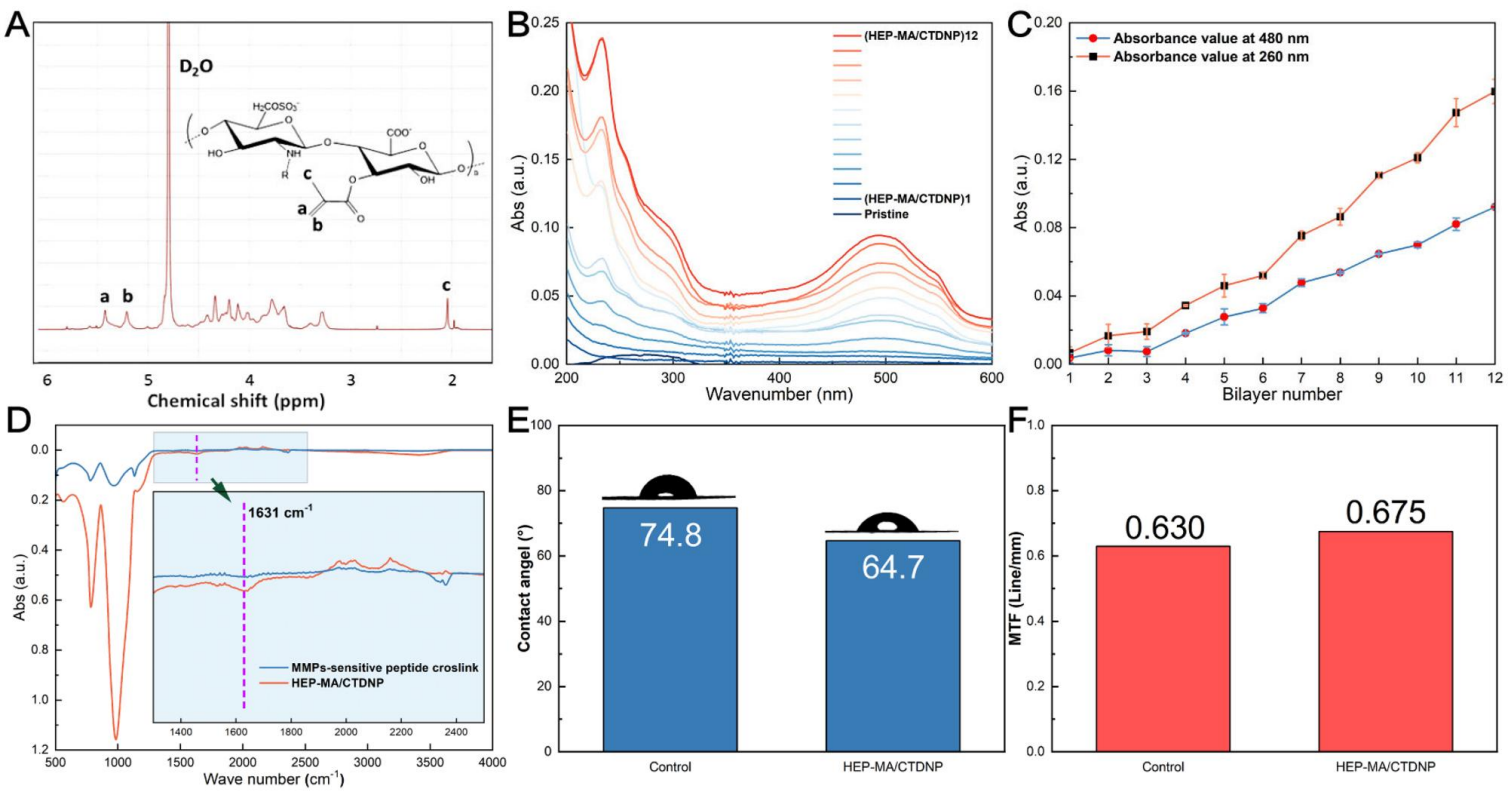
The (HEP-MA/CTDNP) multilayer fabrication and the characterization of multilayered films coated IOL. (**A**) ^1^H NMR spectra of HEP-MA in D_2_O (5 mg mL^−1^). (**B**) The curve of the ultraviolet spectrum during the layer-by-layer assembly process of (HEP-MA/CTDNP)_10_. (**C**) The characteristic UV–Vis absorbance of DOX at 260 and 484 nm increases with HEP-MA/CTDNP multilayer growth. (**D**) FTIR spectra of (HEP-MA/CTDNP)_10_-IOL before and after cross-linking with peptides. (**E**) The contact angle of the material before modification (control) and after modification (HEP-MA/CTDNP). (**F**) MTF of IOL before and after modification.

As shown in Supplementary Figures S1 and S2, the CTDNP was successfully prepared via the polyelectrolyte complexation. The hydrated particle size of the CTDNP, as characterized by the dynamic laser scattering, is around 385 nm (Supplementary Figure S1) and dried particle size, as determined by the scanning electron microscope (SEM), is around 200 nm (Supplementary Figure S2). The HEP-MA was successfully synthesized as well, as indicated by the ^1^H NMR spectrum shown in Figure 2A, where the characteristic hydrogen signal on C=C double bond of methacrylate was shown in the chemical shifts of 5.22 and 5.42 ppm. The nanoparticles and HEP-MA were immobilized onto the IOL surface via layer-by-layer electrostatic assembly. The ultraviolet–visible light spectrometer (UV–Vis) was used to characterize the assembly process (Figure 2B). The nanoparticles were encapsulated with the DOX, as the characteristic absorbance peaks at approximately 260 and 484 nm of DOX was shown in the nanoparticles. As shown in Figure 2C, the drug loading on the IOL surface increases linearly with the deposited layer numbers increasing, indicating that the surface of the IOL is successfully loaded with drugs. The surface morphology of multilayer coatings were also confirmed the immobilization of the nanoparticles (Supplementary Figure S3). Some nanoparticles were observed on the surface, and the surface became progressively smoother with multilayer deposition, which also confirmed the multilayer fabrication (Supplementary Figure S3). GPQG↓IWGQ peptide (↓indicates restriction site) is a typical amino acid sequence sensitive to MMP-2 [37]. The MMP-2 sensitive peptide (GCRD-GPQGIWGQ-DRCG) which contains sulfhydryl groups at both ends was introduced to crosslink the multilayer film, giving the multilayer film with MMP-2 responsive properties. The thiol group of the cysteine residue in the polypeptide chain was crosslinked with the double bond in the (HEP-MA/CTDNP)_10_ film. FTIR was employed to characterize (HEP-MA/CTDNP)_10_ films before and after the peptide cross-linking. As shown in Figure 2D, the un-crosslinked (HEP-MA/CTDNP)_10_ film had a peak at 1631 cm^−1^, which corresponds to the characteristic absorption peak of the C=C double bond in HEP-MA within the multilayer film. This result also shows the successfully synthesized HEP-MA with double bonds. The peak at 1631 cm^−1^ disappeared after cross-linking. The FTIR results confirmed that the reaction between the thiol and HEP-MA double bond of the peptide was successful. The contact angle of the IOL substrate material was 74.8° ± 1.7°. After modification, the contact angle of the surface decreased (64.7° ± 1.0°), which may be related to the introduction of HEP-MA (Figure 2E).

### Optical performance

MTF is an indicator for detecting imaging systems. MTF reverses the contrast change of the image, which is more accurate for judging imaging properties. As an optical material, the optical performance of IOL is very important. In Figure 2F, the MTF of unmodified IOL was 0.63, and the MTF was 0.675 after modification. There was no significant change in MTF before and after modification. They are within the qualified range (≥0.43). The MTF results show that the modified IOL has good optical performance.

### *In vitro* drug release study

**Figure 3.**
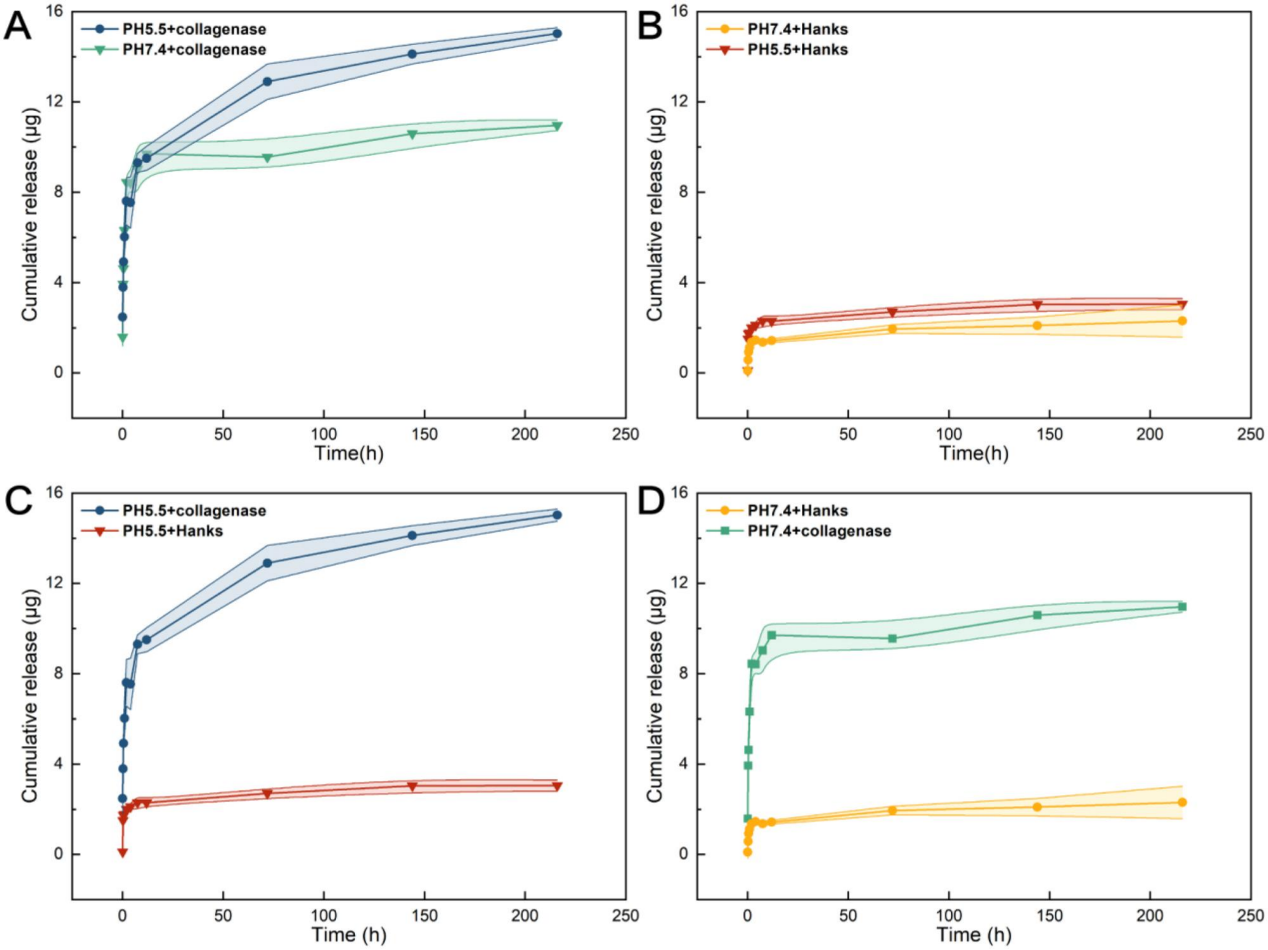
The drug release behavior investigation. *In vitro* cumulative drug release curve from (HEP-MA/CTDNP)_10_-IOL material in different conditions.

Collagenase IV is a member of the MMPs family, which can replace the role of MMP-2 in the *in vitro* experiments. The collected release solution was analysed using fluorescence spectroscopy, and the cumulative release was calculated according to the standard curve. As shown in Figure 3A, in the presence of collagenase, the cumulative drug release of the (HEP-MA/CTDNP)_10_ multilayer film in the environment with a pH of 5.5 is higher than that of an environment with a pH of 7.4. This means that in the presence of collagenase, collagenase IV digested enzyme-sensitive peptides in (HEP-MA/CTDNP)_10_-IOL thereby loosening the multilayer membrane, and the chitosan nanoparticles were protonated in the acidic pathological environment. The DOX in the nanoparticles is released into the solution in the absence of collagenase IV more easily. It can be also seen that the drug loaded in the multilayer film is not released, but the amount of drugs released in the acidic environment was slightly more than that in the physiological environment (Figure 3B). This may be due to that the multilayer film is more stable after being crosslinked by peptides. Therefore, the total amount of drug release from a multilayer film is extremely few. However, in an acidic environment, CHI nanoparticles were protonated, chitosan became soluble. What is more, the DOX may also undergo protonation under acidic conditions, which may also be one of the reasons that the drugs releasing is increased in the acidic conditions. In the case of collagenase, the cumulative release was significantly greater than that in the environment without collagenase, no matter in the acetic condition (Figure 3C) or physiological environment (Figure 3D). These results indicate that the polypeptide crosslinked (HEP-MA/CTDNP)_10_ multilayer film has enzyme-stimulated drug release properties.

### (HEP-MA/CTDNP)_10_-IOL effects on HLEB3 cells

#### *In vitro* cell experiment

**Figure 4.**
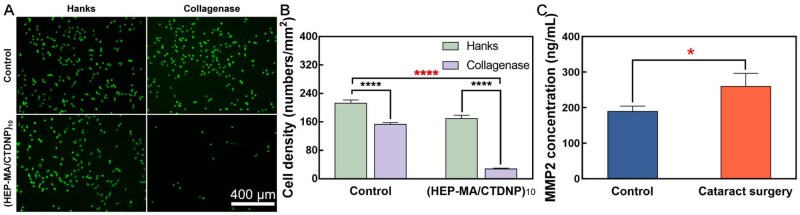
MMP-2 in aqueous humor and the cell responsive behavior to the MMP-2 when on the multilayer coated surface. (**A**) Representative picture of HLEB3 cells on the materials with/without (HEP-MA/CTDNP)_10_ coating; or with/without collagenase IV treatment (2 ng/mL) for 24 h. (**B**) A statistical graph of the number of HLEB3 grown on the surface of IOL material and multilayer film-modified IOL. (**C**) MMP-2 concentration in aqueous humor.

With regards to the cells on the (HEP-MA/CTDNP)_10_-IOL material surface, significant reduction of cell number was found in the presence of collagenase, whereas it showed no significant changes in the absence of collagenase (Figure 4A). However, with regards to the cells on the unmodified IOL material surface, there is no significant difference with and without the collagenase adding. The results showed that after the addition of collagenase, the drug carried in multilayer film was released due to the enzyme loosening the peptide response to multilayer film, leading to the apoptosis of cells. The numbers of cells were calculated and performed statistical analysis. The results showed that the cell count of the modified group after adding the enzyme was statistically different from the other four groups (Figure 4B).

#### Changes of intraocular MMP-2 after cataract surgery

MMPs are a group of important endogenous proteolytic enzymes, which are involved in the ECM remodeling in physiological and pathological processes. They play great part in the degradation and reconstruction of ECM proteins, such as collagen and fibronectin. Degradation of ECM components can promote the migration of LECs, and the reconstruction of ECM is an important pathological mechanism in the development of PCO [38]. Studies have shown that the occurrence of PCO is related to the up-regulation of MMP-2 expression in the eye after cataract surgery. The standard curve of MMP-2 was calculated according to the concentration of the standard substance. The MMP-2 concentration in aqueous humor in the cataract surgery group is 260.75 ± 35.61 ng/mL (Figure 4C). However, the value is 190.34 ± 13.96 ng/mL in aqueous humor in un-operated eyes. The results showed that the two sets of data are statistically different, (*P *= 0.033, *n* = 3, *P *< 0.05). This result confirmed that after cataract surgery, the concentration of MMP-2 in aqueous humor increases compared to un-operative eyes.

#### Subjective PCO evaluation

**Figure 5.**
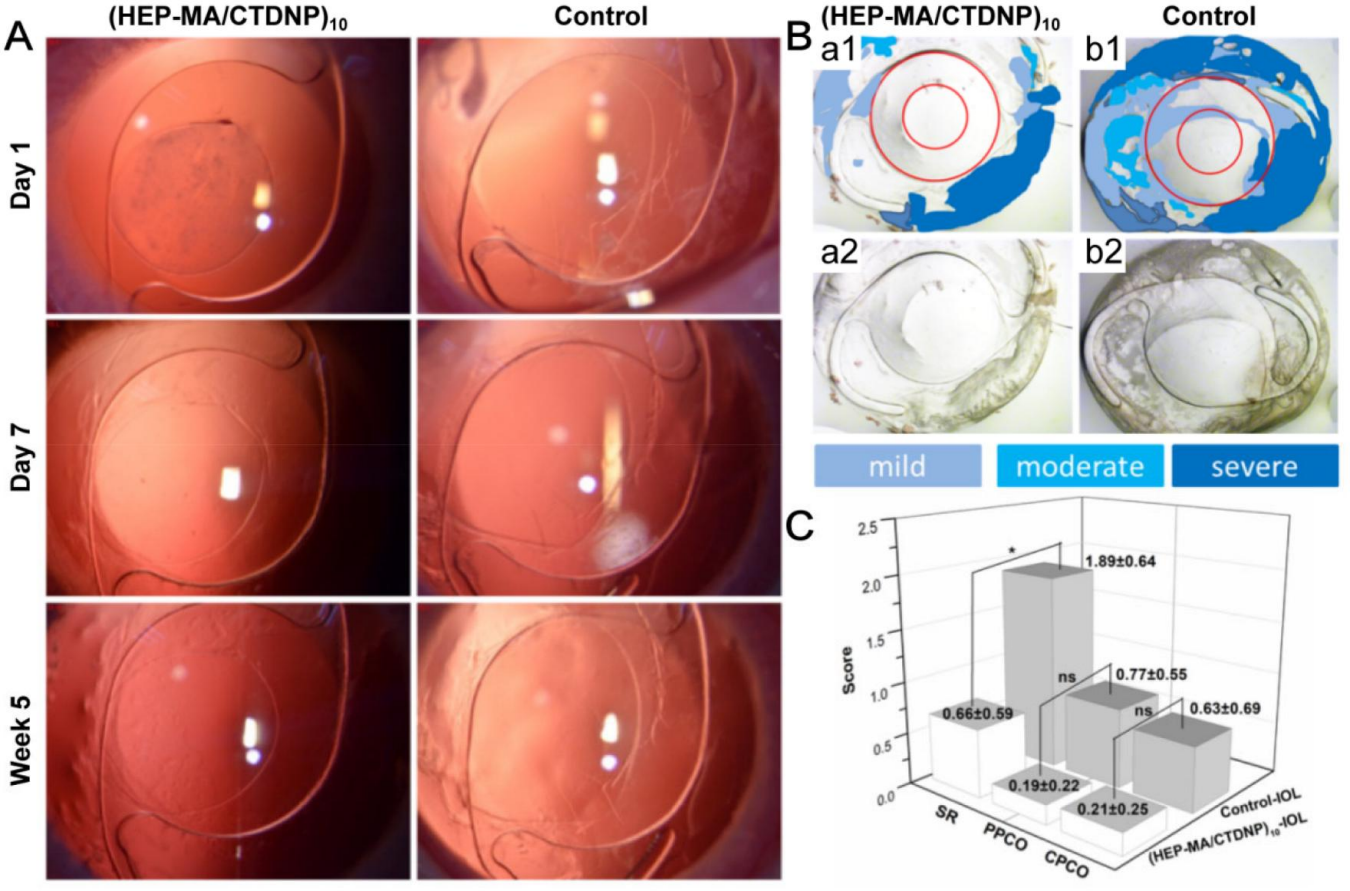
Intraocular IOL implantation and PCO evaluation. (**A**) Slit-lamp micrographs of (HEP-MA/CTDNP)_10_-IOL and Control-IOL in rabbit eye. Images are obtained at 1 day, 7 days and 5 weeks postoperatively. (**B**) Gross photographs from the posterior (Miyake–Apple view) of rabbit eyes implanted with (HEP-MA/CTDNP)_10_-IOL and Control-IOL. (**C**) Statistical analysis of Soemmering’s ring (SR), peripheral PCO (PPCO) and central PCO (CPCO) scores (*means *P *< 0.05, ns means no statistical significance).

All animals survived normally during the entire study period. On the first day after the operation, mild to moderate fibrous exudation of the anterior chamber was observed (Figure 5A). The exudation of the anterior chamber was found in both the experimental and control groups and disappeared after a week. The intraocular exudation of experimental animals after cataract was generally more than the exudation of human eyes, but the exudation in the animal eyes was also absorbed quickly. The degree of PCO was evaluated by slit-lamp observation. There was no obvious difference during 5 weeks observation (Figure 5A). A partially regenerated ECM was seen around the IOL. Because the slit lamp can only observe the optical zone of the IOL, the entire capsular bag was evaluated with a stereomicroscope in the postoperatively fifth week. The area of turbidity is large in the control group, involving the entire optical zone of the IOL and even spread to part of the optical zone, with obvious SR developed. However, in the (HEP-MA/CTDNP)_10_-IOL group, most of the capsule membrane remained transparent, the SR was discontinuous and the regenerated ECM did not progress to the optical zone of the IOL (Figure 5B). PCO is divided into three areas. CPCO is the capsule areas with a diameter of 3.0 mm (red circle inside); PPCO is the capsule areas behind the periphery of the IOL optic (red circle outside); SR is an equatorial region of the capsular bag outside the IOL optic area (Figure 5B). The degree of turbidity of the capsule is marked in different colors according to the severity. The severity of PCO was evaluated by score grading. The score is 0 (none), 1 (slight, iris pattern still detectable), 2 (obvious, iris pattern barely detectable) or 3 (distinct, iris pattern not detectable). Figure 5C shows the PCO scores of the two groups. The control-IOL group showed higher PCO scores (SR: 1.89 ± 0.64, PPCO: 0.77 ± 0.55, CPCO: 0.63 ± 0.69) compared with the (HEP-MA/CTDNP)_10_-IOL group (SR: 0.66 ± 0.59, PPCO: 0.19 ± 0.22, CPCO: 0.21 ± 0.25). The scores of SR, PPCO and CPCO in the (HEP-MA/CTDNP)_10_-IOL group and the control-IOL group were separately analysed by independent sample *t*-test. The *P*-values were 0.03, 0.096, and 0.297, respectively. From the statistical analysis results, it can be seen that the scores of SR are statistically different (*P *< 0.05), but there was no statistical difference in the scores of CPCO and PPCO between the two groups (*P *> 0.05). It can be considered that the (HEP-MA/CTDNP)_10_-IOL group can reduce the occurrence of SR. However, it can be seen from the overall PCO score that the PCO scores in the modified group are lower than those in the control group, indicating that the surface-modified IOL can reduce the severity of PCO (Figure 5C).

#### Objective PCO evaluation

**Figure 6.**
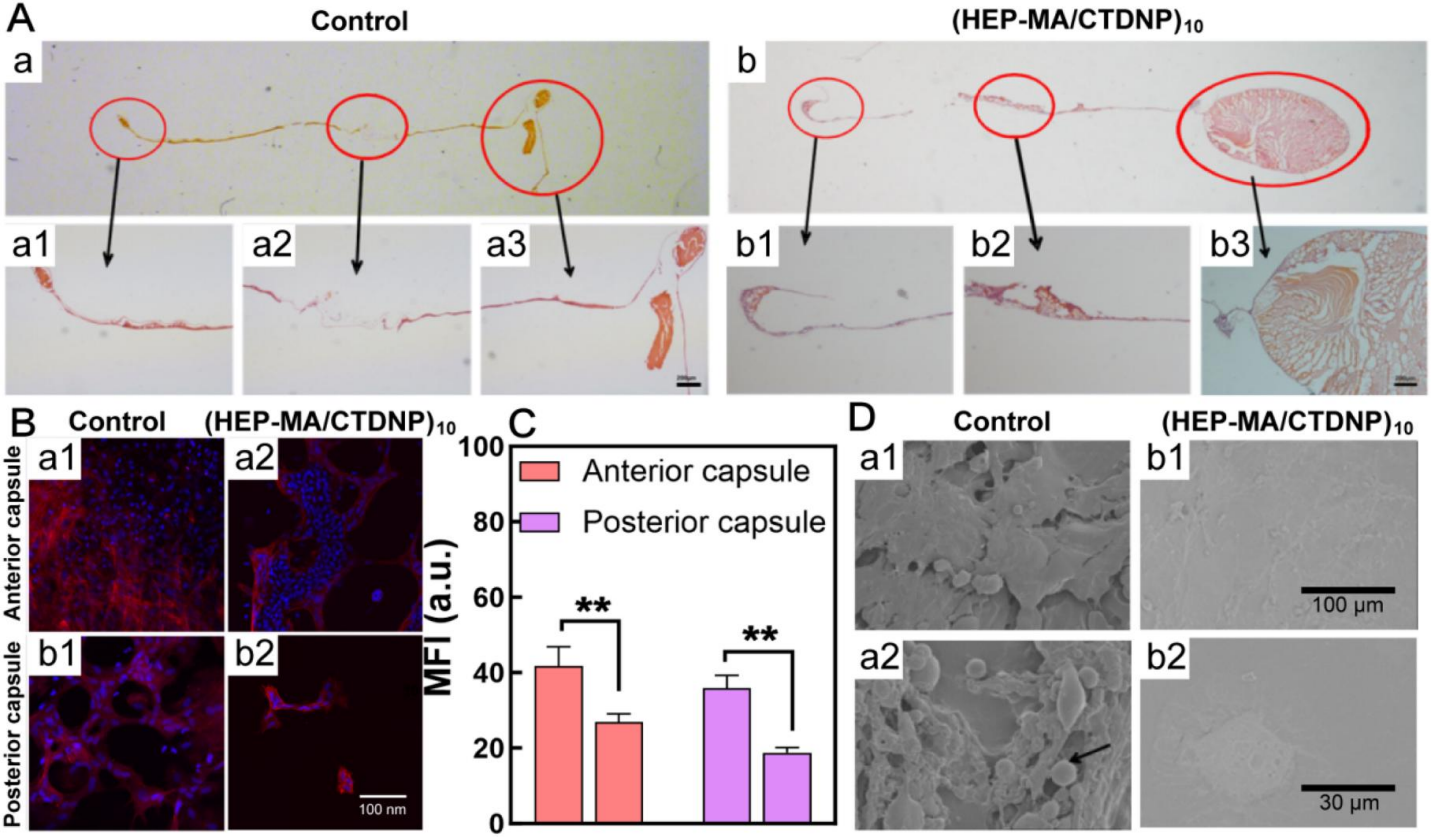
Intraocular IOL implantation and PCO evaluation. (**A**) Representative histological sections of the capsular in (HEP-MA/CTDNP)_10_-IOL group (**A-a**) and Control-IOL group (**A-b**), SR region (**a1, a2, b1, b2**) and CPCO region (**a2, b2**). (**B**) Fluorescence images of posterior capsule (**B-a1, a2**) and anterior capsule (**B-b1, b2**) with rhodamine-labeled phalloidin and DAPI stain. (**C**) The quantitative results of the red fluorescence emitted by phalloidin in **B**. (**D**) SEM image of IOL in the (HEP-MA/CTDNP)_10_-IOL group and the control-IOL group.

Figure 6 is the histological section images of the lens capsule after IOL implantation. It can be seen that SR around the peripheral area of the capsule of the control group was obvious, composed of many cells and ECM (Figure 6A-b1, b3). In addition, the accumulation of cells and ECM was also observed in the center of the capsule (Figure 6A-b2). The few SR and the ECM were found in the (HEP-MA/CTDNP)_10_-IOL group (Figure 6A-a1, a3), with almost no cells found in the central area (Figure 6A-a2). The cytoskeleton deforms during cell migration, the cell arranged in the anterior capsule was disordered in the control groups, cytoskeleton loses its original orderly shape and exhibits the characteristics of migration (Figure 6B-a1). In the (HEP-MA/CTDNP)_10_-IOL implantation group, the cells of the anterior capsule were arranged regularly, and some of the cells were killed and left some cavities (Figure 6B-a2). In the central area of the posterior capsule only a small amount of LECs can be seen (Figure 6B-b2), which is consistent with the HE-stained photograph of the capsule sliced (Figure 6A). As shown in Figure 6B-b1, there are more LECs in the cell-free area in the central area of the posterior capsule, where the shape of the cytoskeleton is relatively spread and the cells are in contact with each other and grow well. The IOL in the capsular bag was taken out in the fifth week to observe the cells on the IOL surfaces. After quantitatively analysing the red fluorescence of the cytoskeleton, it can also be observed that the fluorescence expression of the anterior and posterior capsules in the control group is higher than that in the (HEP-MA/CTDNP)_10_ group (Figure 6C, anterior capsule: *P* = 0.0025; posterior capsule: *P* = 0.0010). This also indirectly confirms that the implantation of the (HEP-MA/CTDNP)_10_-IOL reduces the proliferation of cells within the capsular bag. As shown in Figure 6D, a large amount of substance can be observed on the surface of the control groups. According to the above histological sections, it can be inferred that the material accumulated on the IOL surface is the ECM and LECs (Figure 6D-a1, a2). On the contrary, only a small amount of ECM accumulation and almost no cells were found on the surface of (HEP-MA/CTDNP)_10_-IOL (Figure 6D-b1, b2). These results confirmed that the degree of PCO in the (HEP-MA/CTDNP)_10_-IOL implantation group was lower than that of the control group, indicating that (HEP-MA/CTDNP)_10_-IOL implantation can reduce the progression and severity of PCO.

#### Biocompatibility evaluation

**Figure 7.**
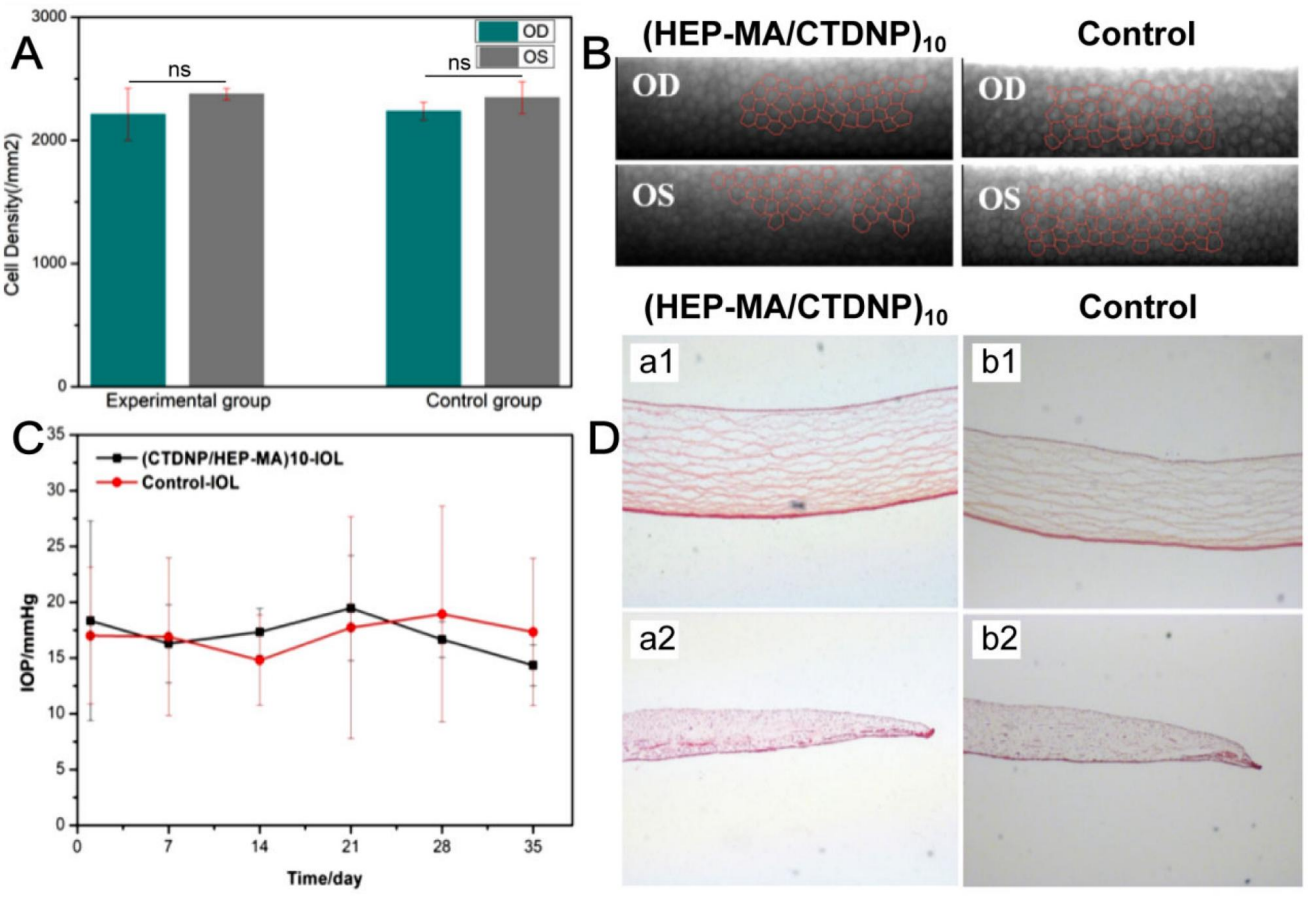
Intraocular IOL implantation and *in vivo* biocompatibility evaluation. The comparison of corneal endothelial cells density (**A**) and morphology (**B**) between oculus dexter (OD, with IOL implantation) and oculus sinister (OS, without IOL implantation) in the (HEP-MA/CTDNP)_10_-IOL group and the control-IOL group. (**C**) Postoperative intraocular pressure in the experimental group and the control group. (**D**) Representative cornea (**a1, b1**) and iris (**a2, b2**) histological section images of the rabbit eyes.

The IOP, tissue morphology of cornea and iris, the corneal epithelium were observed to evaluate the *in vivo* biocompatibility of the (HEP-MA/CTDNP)_10_-IOL (experimental group). The IOLs were implanted in right eyes (OD) and left eyes without surgery were served as the healthy control. Also, the control-IOL were implanted in the control group and left eyes without surgery. It can be seen that there was no obvious abnormality in the density and shape of the cells (Figure 7A and B). The corneal endothelial cells were all regular hexagons and no obvious abnormality. The cell density of the left and right eyes comparison in the experimental group was *P *= 0.054 (>0.05), and in the control group it was *P *= 0.302 (>0.05). There was no statistically difference between the above groups, it can be considered that there is no obvious influence in corneal endothelial cells after implantation (HEP-MA/CTDNP)_10_-IOL. The above results indicated that the morphology, number and structure of corneal endothelium were no obvious abnormality after the surface-modified IOL implantation, indicating that such surface modification is safe. IOP generally increases temporarily after cataract surgery, which may be related to inflammation caused by cataract surgery, destruction of blood humor barrier and incomplete removal of viscoelastic. As shown in Figure 7C, a same trend of postoperative IOP was recorded in both the groups, indicating no significant difference. It can be considered that the modified IOL implantation has no significant effect on IOP. The iris is in front of the lens in the eye, which is the tissue adjacent to the IOL after cataract surgery. Excessive drug concentration in the eye may cause iris congestion or necrosis. The frozen sections of the cornea and iris after (HEP-MA/CTDNP)_10_-IOL implantation are shown in Figure 6D. There was no obvious bleeding or congestion in the tissues, indicating that the modified IOL has good biocompatibility.

## Discussion

MMP-2 expression increased in aqueous humor in the wound healing process post cataract surgery, which is responsible for degrading collagen IV in the basement membrane and ECM. MMPs, which can degrade collagen IV in the basement membrane and ECM, are an indispensable factor involved in cell migration [39]. MMP-2 can degrade collagen and basement membrane collagen IV is involved in the formation of PCO [40]. Previous studies have shown that MMPs are associated with the pathogenesis of anterior capsule opacification and PCO, both of which are caused by the fibrosis of LECs. It is mainly caused by tissue trauma during cataract surgery to stimulate the production of growth factors such as TGF-β [6, 41]. The expression of TGF-β stimulates the interaction of MMP regulatory cells and ECM, and promotes the accumulation of ECM and shrinkage of the lens capsule. Therefore, the expression of MMPs induced by TGF-β is the main driving force of PCO, which may relies on actin cytoskeleton remodeling to facilitate cell invasion and migration [42, 43]. The deformation of the cytoskeleton is one of the signs of cell migration, which is a highly dynamic structural system. Some researchers have proposed that anti-cytoskeleton drugs can inhibit the formation of PCO [44]. It can be seen from the cytoskeleton staining in the control group that the cytoskeleton is highly stretched on the posterior capsule and the pseudopodia of the cells are obvious. Cell movement depends on the crawling of pseudopods, which is closely related to residual cells migrate to the cell-free posterior capsule. The formation of pseudopods depends on the formation of actin microfilament skeletons under the cell membrane, so pseudopods can be clearly observed. In the experimental group, cell cytoskeleton staining in the capsule found that cells had no obvious pseudopodia. On the one hand, the increased expression of MMP-2 in the eye can be used for multilayer film loosening and drug release, on the other hand, after MMP-2 being consumed, it further reduces the occurrence of PCO. The increase MMP concentration in aqueous humor was further confirmed by animal experiments in this research.

PCO is the most common complication following cataract surgery. It can occur between few months and many years after implantation of IOL [6]. The most common treatment for PCO is Nd:YAG laser capsulotomy. However, this may bring new complications, such as IOL injury, retinal detachment and even cystoid macular edema [45]. In order to reduce the possible complications caused by retreatment, it is recommended to modify the preparation directly on the IOL and implant it into the capsular bag together with the IOL to reduce the chance of reoperation. There are various methods of drug delivery, including direct immersion IOL in drugs solution [46], or infusion of the drug into the IOL under high pressure [47]. However, due to the limitation of the preparation method, the quantity of drug load on IOL is limited and the drug release is difficult to control, which is difficult to achieve the expected effect *in vivo*. Therefore, it is necessary to develop drug-loaded IOLs that can have a sustain release property to achieve the purpose of long-term inhibition of PCO. In addition, studies have also found that the existing drug treatments, especially the use of anti-proliferative drugs are often indiscriminate. They kill the target cells and also cause damage to adjacent normal cells, and the burst release of drugs can also cause serious toxic and side effects. Therefore, it is particularly important to design and develop safe drug release coatings for surface modification of IOLs. So, in this experiment, we designed a stimulus-responsive drug release smart drug carrier coating, which can release drugs responsively under the stimulation of MMP-2.

The GCRD-GPQGIWGQ-DRCG polypeptide chain is a commonly used polypeptide which is sensitive to MMP-2, in order to improve the safety of drug-loaded IOL and further control the release of drugs, we introduced MMP-2 stimulating factors and designed a drugs sustained-release IOL which responds to MMP-2. It is a perfect *in situ* drug carrier due to the characteristics of IOL [48]. After implantation in the eye, the IOL and the capsule are in close contact, and the remaining cells are directly attached to the IOL. We found that the proliferating cells and ECM are not only on the capsule membrane, but also on the surface of the IOL in our previous researches. So the anti-adhesion performance of the IOL is also very important. Therefore, in this study, we added hydrophilic heparin sodium to endow the multilayer film hydrophilic and anti-adhesion properties. In the reconstruction process of the multilayer film, in order to enhance the drug elution performance and give the multilayer membrane enzyme-responsive performance, the HEP-MA in the multilayer film interacts with the MMP-2 sensitive polypeptide GCRD-GPQGIWGQ-DRCG undergoes Michael addition reaction to strengthen the stability of the multilayer film. The principle of the cross-linking of multilayer films by polypeptides is the reaction between the residual thiol group of the cysteine and the double bond in HEP-MA through the thiol-olefin. On the other hand, nanomedicine is an effective way for disease treatment [49]. Several nanoparticles have been developed in the ophthalmic diseases treatments [50–53]. CHI has good biocompatibility and has passed the US Food and Drug Administration approval of biological materials. As a natural polymer and cheap biological material, it is a promising auxiliary material. Pharmaceutical industry uses it in various formulations such as controlled drug delivery systems, wound dressings, hemostatic sponges, tissue engineering scaffolds or space filling implants [54]. CHI is also a suitable candidate for ophthalmic formulations due to its biodegradability, non-toxicity, antibacterial and antifungal effects. Therefore, we choose chitosan as a nan-carrier to encapsulate drugs. The main advantage of using nanoparticles is that they can enhance the bioavailability and achieve a controlled release result, targeted delivery, and ultimately improve the therapeutic effect. In addition, chitosan also has a high antibacterial ability due to the polycation structure, which can combine with negatively charged bacterial cells, thereby destroying cells and bacterial membranes and causing bacterial cell death [55]. Chitosan nanoparticles combined with MMP-2 sensitive polypeptide and HEP-MA cross-linking in the multilayer film to strengthen the stability of drug release performance.

A balance is needed between effectiveness and safety, and the research on drug sustained-release IOLs needs to be followed up to ensure that the effectiveness is improved while ensuring safety. IOL as a foreign body implanted in the eye can cause anterior chamber inflammation, coupled with the destruction of the blood humor barrier after the operation, followed with IOP increasing. There was no significant difference in IOP between the experimental group and the control group, showing that the modified IOL has good biocompatibility. Corneal endothelial cells are a single layer of regular hexagonal cells located behind the posterior elastic layer of the cornea. They play a role in maintaining the normal function of the cornea. Corneal endothelial cells maintain the thickness of the cornea through active fluid pumping and mechanical barrier function. The basic function of corneal endothelial cells is very important for cornea transparency. If the density of corneal endothelial cells decreases, or its damage exceeds its compensatory capacity, the sodium pump and barrier function are destroyed, and the permeability of the endothelium increases, leading to corneal edema. Since the number of corneal endothelium is related to age and weight, there are also differences between individuals, so we compare the left and right eyes. The corneal endothelium results showed that the corneal endothelium of the experimental eye and the contralateral eye had no significant difference in shape and quantity. However, on the whole, the number of corneal endothelium in the experimental eye is less than that of the contralateral eye without IOL implantation. Although the function and operation skills of surgical instruments during cataract surgery have been improved recently, and the protection of viscoelastics during surgery, it is still inevitable to cause damage to the corneal endothelium, but this damage is slight and does not affect the normal function of the corneal endothelium. Iris and corneal is in the anterior chamber before the lens, so it is an indicator to detect the biocompatibility of the modified IOL. There is no obvious bleeding or congestion in the iris and corneal tissue, indicating a good biocompatibility after modification. After animal implantation (HEP-MA/CTDNP)_10_-IOL, compared with the control group implanted with unmodified IOL, the severity of PCO was reduced.

Although the intelligent drug-release coating IOL that responds to MMP-2 developed in this study shows potential in inhibiting posterior capsular opacification (PCO), there are still some limitations. Currently, it is mainly based on animal experiments, and its safety and effectiveness in humans need to be verified through large-scale clinical trials. The drug release is affected by the ocular microenvironment, making it difficult to precisely control.

Future research plans include conducting long-term animal experiments on multiple species and human clinical trials, optimizing the material and drug systems, and improving the preparation process. However, considering the urgent clinical need for new methods of preventing PCO, this technology integrates multidisciplinary innovations. It not only has the potential to become a routine choice for cataract surgery but can also be extended to the treatment of other ocular diseases. Moreover, with the increase in the number of cataract patients, after its industrialization, it has a broad market prospect and can promote the technological upgrading of the ophthalmic medical industry.

## Conclusion

In summary, MMP-2 and pH sensitively drug releasing coating were designed and coated onto IOL surface to improve the PCO prevention. The *in vitro* release study results show that the prepared surface-modified IOL has the most cumulative drug release under the condition of the enzyme. It shows that the surface-modified IOL has the function of enzyme responsibility to control the drug release. In the intraocular implantation experiment, the PCO degree of the surface-modified IOL group was lower than that of the control-IOL group, which is consistent with the results of the stereomicroscope observation. In the histological section of the lens capsule, it was found that the SR on the capsular bag periphery in the unmodified IOL implantation group was more obvious. The degree of PCO in the surface-modified IOL group was lower than that in the control group. It can also be observed that such surface-modified IOL has good *in vivo* biocompatibility, as it does not cause abnormal to the IOP, adjacent tissues and cells. These results indicate that the MMP-2 and pH sensitively drug releasing coating-modified IOL can inhibit PCO progress, and has better biocompatibility.

## Supplementary Material

rbaf077_Supplementary_Data
