# Supplementary material for: Matrix metalloproteinase-2 and pH-responsive drug eluting multilayer as intraocular lens coating to improve the posterior capsule opacification inhibition
Source: Regen Biomater. 2025 Jul 28;12:rbaf077. doi: 10.1093/rb/rbaf077 (PMC12364434; doi:10.1093/rb/rbaf077)
Supplement: rbaf077_Supplementary_Data [file rbaf077_supplementary_data.docx]

Supplementary Information

**MMP-2 and pH-responsive drug eluting multilayer as IOL coating to improve the PCO inhibition**

National Engineering Research Center of Ophthalmology and Optometry, School of Biomedical Engineering, School of Ophthalmology and Optometry, Eye Hospital, Wenzhou Medical University, Wenzhou 325027, China

*Correspondence author: [linqk@wmu.edu.cn](mailto:linqk@wmu.edu.cn)

**Experimental Section**

**Materials**

Chitosan (CHI, molecular weight (MW) around 179.17 kDa, high viscosity (>400 mPa.s)) and TPP were purchased from Aladdin Biochemical Technology Co., Ltd. (Shanghai, China). Doxorubicin hydrochloride (DOX) was purchased from Meilun Biotechnology Co., Ltd. (Dalian, China). N-hydroxysulfosuccinimidebsodium (NHS) and N-(3-dimethylaminopropyl)-N’-ethylcarbodiimide hydrochloride (EDC) were purchased from Sigma-Aldrich (St Louis, MO, USA).

**Measurements**

**CTDNP Nanoparticles Fabrication and Characterization**

At room temperature, a 0.2% (w/v) aqueous solution of TPP and 0.05% (w/v) of DOX were gradually introduced drop by drop into a 0.1% CHI solution (dissolved in 1% acetic acid at a w/v concentration) while being magnetically stirred at 500 revolutions per minute. Subsequently, the drug-loaded nanoparticles (termed CTDNP for short) were chemically cross-linked with 40 mM of EDC and 20 mM of NHS at room temperature for 1 hour.

The particle size of the nanoparticles was gauged by dynamic light scattering, making use of an Autosizer 3000 (manufactured by Malvern Instruments Limited, UK). Each sample was measured three times to calculate the Z-average particle size and the average zeta potential. The morphology of the nanoparticles with an N/P ratio of 2.5 was visualized using a scanning electron microscope (SEM, produced by Phenom-World, the Netherlands). The N/P ratio denotes the molar ratio of the nitrogen present in the repeating unit of CHI to the phosphorus in TPP.


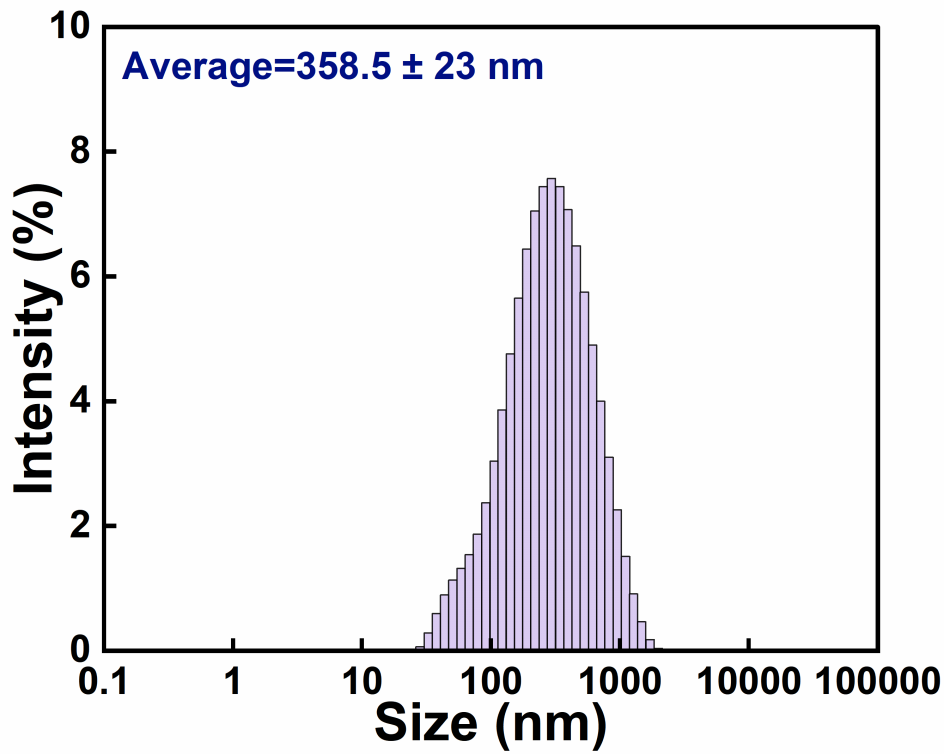


**Figure S1 The particle size of CTDNP.**


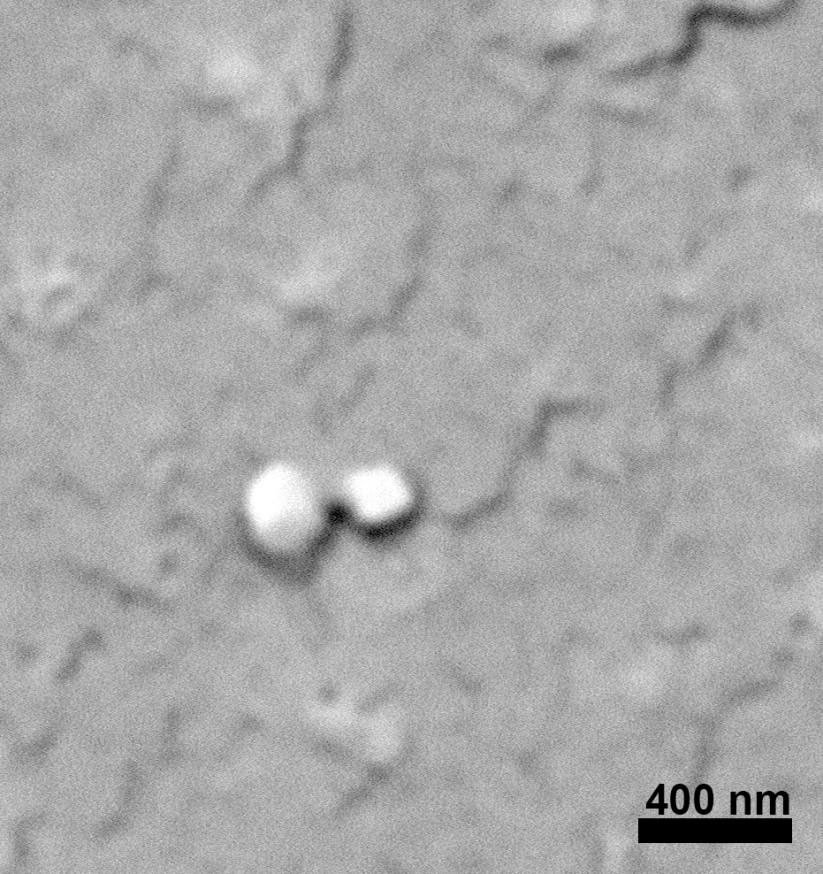


**Figure S2 The SEM image of CTDNP.**

**
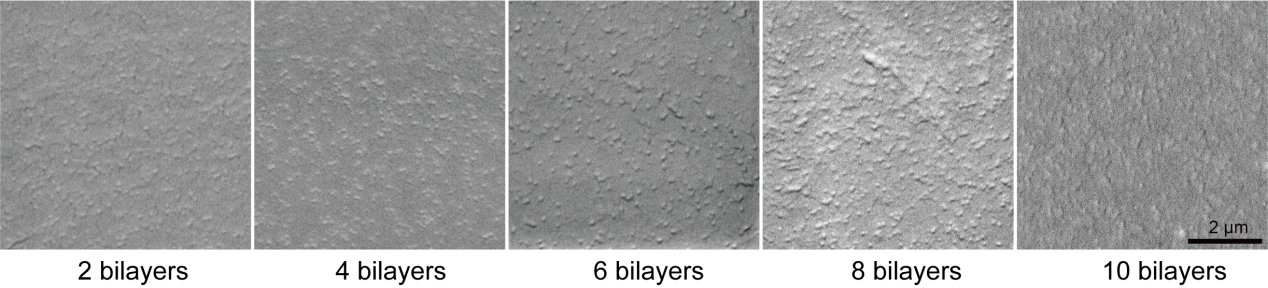
**

**Figure S3 The SEM image of the multilayer surface.**
